# Neurocognitive Outcome and Seizure Freedom After Awake Surgery of Gliomas

**DOI:** 10.3389/fonc.2022.815733

**Published:** 2022-04-07

**Authors:** Sarah Christina Reitz, Marion Behrens, Irina Lortz, Nadine Conradi, Maximilian Rauch, Katharina Filipski, Martin Voss, Christian Kell, Marcus Czabanka, Marie-Therese Forster

**Affiliations:** ^1^ Department of Neurosurgery, University Hospital Frankfurt, Goethe University, Frankfurt/Main, Germany; ^2^ Department of Neurology, University Hospital Frankfurt, Goethe University, Frankfurt/Main, Germany; ^3^ Epilepsy Center Frankfurt Rhine-Main, Center of Neurology and Neurosurgery, University Hospital Frankfurt, Goethe University, Frankfurt/Main, Germany; ^4^ Institute of Neuroradiology, University Hospital Frankfurt, Goethe University, Frankfurt/Main, Germany; ^5^ Edinger Institute, Institute of Neurology, University Hospital Frankfurt, Goethe University, Frankfurt/Main, Germany; ^6^ University Cancer Center Frankfurt (UCT), University Hospital Frankfurt, Goethe University, Frankfurt/Main, Germany; ^7^ German Cancer Consortium (Deutsches Konsortium für Translationale Krebsforschung), Partner Site Frankfurt/Mainz, Heidelberg, Germany; ^8^ German Cancer Research Center (Deutsches Krebsforschungszentrum), Heidelberg, Germany; ^9^ Dr. Senckenberg Institute of Neurooncology, University Hospital Frankfurt, Goethe University, Frankfurt/Main, Germany

**Keywords:** glioma, neurocognitive outcome, quality of life, epilepsy, neurocognition, awake surgery

## Abstract

**Objectives:**

Gliomas are often diagnosed due to epileptic seizures as well as neurocognitive deficits. First treatment choice for patients with gliomas in speech-related areas is awake surgery, which aims at maximizing tumor resection while preserving or improving patient’s neurological status. The present study aimed at evaluating neurocognitive functioning and occurrence of epileptic seizures in patients suffering from gliomas located in language-related areas before and after awake surgery as well as during their follow up course of disease.

**Materials and Methods:**

In this prospective study we included patients who underwent awake surgery for glioma in the inferior frontal gyrus, superior temporal gyrus, or anterior temporal lobe. Preoperatively, as well as in the short-term (median 4.1 months, IQR 2.1-6.0) and long-term (median 18.3 months, IQR 12.3-36.6) postoperative course, neurocognitive functioning, neurologic status, the occurrence of epileptic seizures and number of antiepileptic drugs were recorded.

**Results:**

Between 09/2012 and 09/2019, a total of 27 glioma patients, aged 36.1 ± 11.8 years, were included. Tumor resection was complete in 15, subtotal in 6 and partial in 6 patients, respectively. While preoperatively impairment in at least one neurocognitive domain was found in 37.0% of patients, postoperatively, in the short-term, 36.4% of patients presented a significant deterioration in word fluency (p=0.009) and 34.8% of patients in executive functions (p=0.049). Over the long-term, scores improved to preoperative baseline levels. The number of patients with mood disturbances significantly declined from 66.7% to 34.8% after surgery (p=0.03). Regarding seizures, these were present in 18 (66.7%) patients prior to surgery. Postoperatively, 22 (81.5%) patients were treated with antiepileptic drugs with all patients presenting seizure-freedom.

**Conclusions:**

In patients suffering from gliomas in eloquent areas, the combination of awake surgery, regular neurocognitive assessment - considering individual patients´ functional outcome and rehabilitation needs – and the individual adjustment of antiepileptic therapy results in excellent patient outcome in the long-term course.

## Introduction

Gliomas are the most frequent malignant primary brain tumors, with an incidence of 7.1 per 100 000 persons/year (1). Most common clinical manifestation of low grade gliomas are epileptic seizures, whereas patients with high grade gliomas additionally often suffer from neurologic deficits at the time of diagnosis of the tumor (2–4).

One of the major determinants of quality of life in glioma patients is neurocognitive functioning (5). Seizures as well as cognitive symptoms affecting higher cerebral functions (e.g. attention, memory, communication, executive functions) may have great impact on patients’ daily life, including their neuropsychological wellbeing (6).

Current standard of therapy is maximal tumor resection followed by adjuvant therapy (7, 8). Especially tumors located in “eloquent” areas need to be resected with utmost care. In order to optimize the neurologic and simultaneously oncological outcome of these patients, awake surgery is the method of choice to balance maximal extent of tumor resection (EOR) with preservation of neurologic function (9–11).

Several studies focusing on patients´ neurologic and neurocognitive outcome after awake surgery have been published during recent years (10, 12, 13). However, reports on patients suffering from glioma in language-related localizations as well as longitudinal long-term follow-up evaluations on patient’s neurocognitive performances beyond 6 months after surgery are scarce.

We therefore aimed at evaluating neurocognitive functioning in patients suffering from gliomas located in language-related areas before and after awake surgery as well as during their follow up course of disease. We assessed changes in patients´ neurocognitive functioning across different time points of the disease as well as epileptic seizure occurrence. Such information could be of clinical relevance to refine patients neurocognitive monitoring on an individual basis.

## Methods

### Study Design

We performed a prospective single-center study in patients who underwent awake surgery of glioma located in language-related areas of the dominant hemisphere between 09/2012 and 09/2019 in our department. Patients underwent neuropsychological evaluation as part of their pre-surgical work-up, as well as during the follow up of their disease. Patients´ clinical characteristics as well as data on seizure outcome were recorded at each follow-up visit.

Study approval was granted by the local Ethics Committee (SNO 08/2016). All procedures performed were in accordance with the ethical standards of the institutional research committee and with the standards laid down in the Declaration of Helsinki (14). All patients gave written informed consent prior to data collection.

### Patients

During the above-mentioned period, all patients meeting the criteria for study inclusion were identified. Inclusion criteria comprised (1) adult patients aged ≥ 18 years (2), tumor localization in language-related areas, i.e. the inferior frontal gyrus (IFG), the anterior temporal lobe (ATL), the dorsal superior and middle temporal gyrus and the supramarginal gyrus (dMTG/STG), in the language-dominant hemisphere (language-dominance was determined by fMRI) (4), left hemispheric dominance (5), fluent knowledge of German and, thus (6), indication for awake tumor surgery. Regarding exclusion criteria these were (1) age ≤ 18 years (2), right hemispheric dominance (3), other tumor locations as indicated above as well as general exclusion criteria for awake craniotomy such as (4) severe language deficits to the extent of clinically relevant aphasia at tumor diagnosis as well as (5) only sparse knowledge of German, English or French. Indication for surgical treatment as well as postoperative treatment was recommended by a multidisciplinary tumor-board for each patient.

Awake tumor resection was performed employing awake mapping and monitoring techniques to allow for intraoperative testing and preservation of speech function, in addition to motor or sensory evoked potential monitoring. In detail, an asleep-awake-asleep technique was employed. Brain mapping was performed using bipolar stimulation applying a frequency of 50Hz and a stimulation intensity of 3 to 6 mA once the patient was awake before tumor resection. After cortical mapping tumor resection was begun with regard to functional boundaries, repeating electrical stimulation at intervals during subcortical preparation. Language tasks comprised counting and naming in all patients, while reading, word and sentence comprehension, calculation and repetition tasks were used according to the location of the tumor (15).

Early postoperative MRI to assess EOR was performed within 72 hours after surgery in all patients. EOR and tumor progression were evaluated by a board-certified neuro-radiologist, with EOR being defined as complete (gross-total tumor resection; GTR), subtotal (STR; with less than 10% of the original volume as residual tumor) or partial (PR; with residual tumor coming up to more than 10% of the original volume), and disease progression being determined according to the RANO criteria (16). Brain tumor diagnoses were assigned according to the 2016 *WHO Classification of Tumors of the Central Nervous System* (17) (4^th^ and 4^th^ revised version, respectively, according to the year of inclusion into the study). Formalin-fixed paraffin-embedded tumor tissue sections were mounted on slides, H&E-stained following established protocols and evaluated by an experienced neuropathologist (KF). For IDH mutation analysis tissue sections were stained with a mutation specific antibody against IDH1_R132H (clone H09, Dianova, Hamburg, Germany). Representative tumor regions with highest cancer cell ratios were selected for punch biopsy or 4-10x10 µm whole slide tumor tissue collection and further molecular pathological analysis. Tumors from patients included since 2017 were subjected to large-scale DNA methylation analysis by use of the Illumina EPIC Human Methylation array (Illumina, California, USA) after DNA isolation. Patients´ clinical characteristics, including the Karnofsky-Performance score (KPS), data on seizure outcome, and results of magnetic resonance imaging were recorded at regular, usually three-months, follow-up visit.

Occurrence of epileptic seizures (seizures yes/no) and number of antiepileptic drugs (AED) were retrospectively assessed on basis of the electronic patient file timed to the neurocognitive assessments (median time difference between the evaluation of postoperative epileptic seizures and patients´ neurocognitive performances: t2, 0.4 (IQR 0-1.6) months; t3, 0.7 months, IQR 0-1.9).

### Neurocognitive Assessment

Neurocognitive assessment was performed at three different time points; as part of the preoperative work-up (t1), at follow up <9 months (median 4.1 months, IQR 2.1-6.0) after surgery (t2) and at follow up >9 months (median 18.3 months, IQR 12.3-36.6) after surgery (t3). While neurocognitive performance was evaluated in all patients before surgery, data on postoperative assessments had to remain incomplete, either due to patients’ non-compliance or due to refusal to undergo further evaluation. Thus, follow-up evaluations at time points t2 and t3 were conducted in a subset of 23 patients and 20 patients, respectively. As a result, complete longitudinal neurocognitive assessment with evaluations at all three time-points was possible in 16 patients.

Each assessment was performed by a trained neuropsychologist and took patients approximately 1.5 h to complete. The applied test-battery included tests for attention, verbal fluency, verbal memory, figural memory, working memory, executive functioning, visuospatial functioning, as well as the assessment of emotion such as anxiety and depression. A z-score <-1.5 was defined as the cut-off for the definition of an impairment, a change in z-score > ± 1 was defined as the cut-off for a significant change in cognitive performance. A detailed list of all tests is provided in **Table 1**.

**Table 1:** Neurocognitive assessment, tasks per neurocognitive domain.

Cognitive domain	Test	Cognitive function
**Attention**	TAP Alertness (18)	Response time
	TAP Geteilte Aufmerksamkeit II (18)	Divided attention
**Verbal fluency**	Wortschatztest (WST) (19)	Vocabulary (passive)
	Regensburger Wortflüssigkeitstest (RWT) (20)	Verbal fluency (active)
**Verbal memory**	Wechsler Memory Scale – Revised (WMS-R) (21)	Verbal memory span
	Verbaler Lern- und Gedächtnistest (VLMT) (22)	Verbal short- and long-term memory
**Figural memory**	Benton Test (23)	Figural short-term memory
	Rey–Osterrieth complex figure test (ROCFT) (24)	Figural long-term memory
**Working memory**	Wechsler Memory Scale – Revised (WMS-R) (21)	Verbal working memory
	TAP Arbeitsgedächtnis (18)	Verbal working memory
**Executive functioning**	Verbaler Lern- und Gedächtnistest (VLMT) (22)	Interference
	TAP Inkompatibilität (18)	Inhibitory control
	Leistungsprüfsystem (LPS) (25)	Reasoning
	Tower of London (ToL) (26)	Problem solving/planning
**Visuospatial functioning**	Rey–Osterrieth complex figure test (ROCFT) (24)	Visual-spatial ability
**Mood**	Beck Depressionsinventar 2 (BDI-II) (27)	Depression
	Beck Angstinventar (BAI) (28)	Anxiety

### Statistical Analysis

Regarding baseline characteristics, values are presented as numbers with percentages and medians with inter quartile range (IQR) or means (depending on the presence of normal-distribution, tested by quantile-quantile plots), unless otherwise indicated.

Comparing dependent binary variables (impairment yes/no) at different timepoints Cochrans Q Test was used, for *post-hoc* tests McNemar Test was performed. Comparing continuous interval scaled variables (z-values) dependent samples student-t-Test was used. The significance level was set to p<.05.

Statistical analysis was performed with SPSS 26.0 for Windows (2019, IBM Corp.; Armonk, NY) and GraphPad Prism 9.0 for MacOS (2021, GraphPad Software, La Jolla CA). Measurement of tumor resection volume was performed using the SmartBrush tool of the Brainlab Elements software (Brainlab AG, Munich, Germany).

## Results

The study cohort comprised 27 patients meeting the inclusion criteria. Patient baseline characteristics are listed in **Table 2**.

**Table 2:** Demographic data.

Characteristics	^1^number (percentage), ^2^median (IQR), ^3^mean (standard deviation)
**Gender, female**	10 (37%)^1^
**Age, years**	36.1 (11.8)^3^
**Education, years**	13 (10-13)^2^
**Left hemispheric dominance**	27 (100%)^1^
**Histology**	
Astrocytoma	15 (55.6%)^1^
Oligodendroglioma	8 (29.6%)^1^
Glioblastoma	4 (14.8%)^1^
**WHO grade**	
I	2 (7.4%)^1^
II	6 (22.2%)^1^
III	15 (55.6%)^1^
IV	4 (14.8%)^1^
**IDH mutation**	19 (70.4%)^1^
**Tumor location**	
IFG	16 (59.3%)^1^
ATL	4 (14.8%)^1^
dMTG/STG	7 (25.9%)^1^
**Preoperative tumor volume, cm^3^**	15.3 (7.5-37)^2^
**EoR**	
100% (GTR)	15 (55.6%)^1^
90-99% (STR)	6 (22.2%)^1^
<90% (PR)	6 (22.2%)^1^
**Adjuvant treatment**	
Combined radiochemotherapy	21 (77.8%)^1^
No adjuvant treatment	6 (22.2%)^1^

Data is presented as ^1^number (percentage), ^2^median (IQR) or ^3^mean (standard deviation). WHO (world health organization), EoR (extent of resection), IFG (inferior frontal gyrus), ATL (anterior tempral lobe), dMTG/STG (dorsal medial and superior temporal gyrus/supramarginal gyrus), GTR (gross total resection), STR (subtotal resection), PR (partial resection).

All patients had a left hemispheric dominance according to the Edinburgh Handedness Inventory (29). Gliomas were all located in the left hemisphere involving the inferior frontal gyrus, the anterior temporal lobe or the dorsal superior and/or medial temporal gyrus in 16, 4 and 7 patients, respectively. Tumor histology revealed astrocytoma in 15 patients, oligodendroglioma in 8 patients and glioblastoma in 4 patients. IDH mutation was present in 19 patients. Early postoperative MRI revealed that complete (gross total resection, GTR) or subtotal tumor resection (STR) could be achieved in 15 and 6 (55.6% and 22.2%) patients, respectively, while partial resection (PR) could only be achieved in 6 (22.2%) patients. Of note, tumor resection had been stopped as soon as a patient experienced speech function worsening beyond slight semantic or phonological paraphrasia or if a patient had got too tired to perform the respective tasks allowing safe tumor resection without harming speech function. During the first days after surgery 13 patients suffered from transient slight aphasia.

As a consequence, as well as with regard to patients´ postoperative neurocognitive performance, 17 (63%) patients underwent postoperative rehabilitation therapy in highly-specialized neurological rehabilitation hospitals. All other patients were recommended to undergo individual physical, neurocognitive, linguistic and/or occupational therapy in an outpatient setting.

Following tumor board recommendation and patients’ personal preference, 21 (77.8%) patients received adjuvant therapy - either after in-patient or during out-patient rehabilitation therapy - with concomitant radio-chemotherapy, and 6 (22.2%) patients were treated only surgically, without adjuvant treatment.

### Outcome and Epileptic Seizures

After the first follow-up period of 18.3 months (t2), 21 (91.3%) patients showed stable disease, presenting a median KPS of 100% (IQR 90-100). Most importantly, in the long-term follow-up (t3), stable disease was still diagnosed in 19 (70.4%) patients, and their median KPS came up to 100% (IQR 90-100).

Regarding epileptic seizures, prior to surgery, 18 (66.7%) patients suffered from epilepsy, of which 8 patients had generalized tonic-clonic epileptic seizures, 10 patients had focal seizures. 20 patients reported a regular intake of at least one AED. At the last visit (t3) no patient suffered from ongoing epileptic seizures, thus 100% of the patients corresponded to an Engel Class 1 according to the ILAE classification (30). Thus, significant decrease in the occurrence of epileptic seizures was observed after glioma treatment, comparing seizure activity at baseline (t1) and at patients´ last visit (n=27, time between visit and surgery median 15.9 months, IQR 7.5-34.2; χ²=27.0, p<0.001). However, at this time point, 22 (81.5%) patients still reported on the intake of at least one AED (1 AED 66.7%, 2 AED 14.8%; z=-.894, p=.371). **Table 3** gives an overview about the outcome.

**Table 3:** Outcome data.

			t1 n=27	t2 n=22	t3 n=20	last visit n=27
**KPS (median, IQR)**			100 (100-100)	100 (90-100)	100 (90-100)	100 (90-100)
**MRI, SD, n (%)**				21 (91.3%)	19 (95.0%)	25 (92.6%)
**Seizures, yes, n (%)**			18 (66.7%)	2 (8.7%)	0 (100%)	0 (100%)
**Number of AEDs, n (%)**						
0			7 (25.9%)	3 (13.0%)	4 (20.0%)	5 (18.5%)
1			18 (66.7%)	14 (60.9%)	13 (65.0%)	18 (66.7%)
≥2			2 (7.4%)	6 (26.1%)	3 (15.0%)	4 (14.8%)

Data is presented as number (%) or if marked as median (IQR).

Timepoints: t1 (preoperatively), t2 (after a median follow-up period of 4.1 months) and t3 (after a median follow-up period of 18.3 months). The fourth column (“last visit”) considers the last available visit for each patient (n=27, time between visit and surgery median 15.9 months). KPS (Karnofsky performance score), MRI (magnetic resonance imaging), SD (stable disease), AED (antiepileptic drugs).

### Neurocognitive Performance – Number of Impairments

Prior to surgery, impairment in at least one neurocognitive domain was found in 37% of patients. With deterioration of 22% of patients working memory was the most frequently impaired domain. Mood disturbances were observed affecting 66.7% of patients.

With regard to significant changes in the number of cognitively impaired patients over time these were found for the domains verbal fluency (Cochran’s Q (14)=9.33, p=.009), executive functioning (Cochran’s Q (15)=6.0, p=.049) and mood (Cochran’s Q (15)=7.0, p=.030).

In detail, for the domain verbal fluency, *post-hoc* tests showed that the number of impaired patients increased significantly from t1 to t2 (p=.031). At t3, this number had decreased nearly to baseline, however, this change did not reach the level of significance (t3 vs. t2, p=.125; t3 vs t1, p=.5; t1 7.7% impairment vs. t3 10.5% impairment).

Although for executive functioning similar tendencies were observed, *post-hoc* analysis found no significant changes between t2 and t1 (p=.219) and between t3 and t1 (p=1.0), and only a trend for improvement between t3 and t2 (p=.063). Nevertheless, at t3, impairment in executive functioning was observed in only 5% of patients compared to 14.8% of patients preoperatively.

As mentioned, patients´ mood was especially affected prior to surgery (t1 66.7%). However, *post-hoc* tests showed a trend for improvement comparing t1 and t2 (p=.07), with at t3 only 35% of patients presenting mood disturbances.

**Figure 1:**
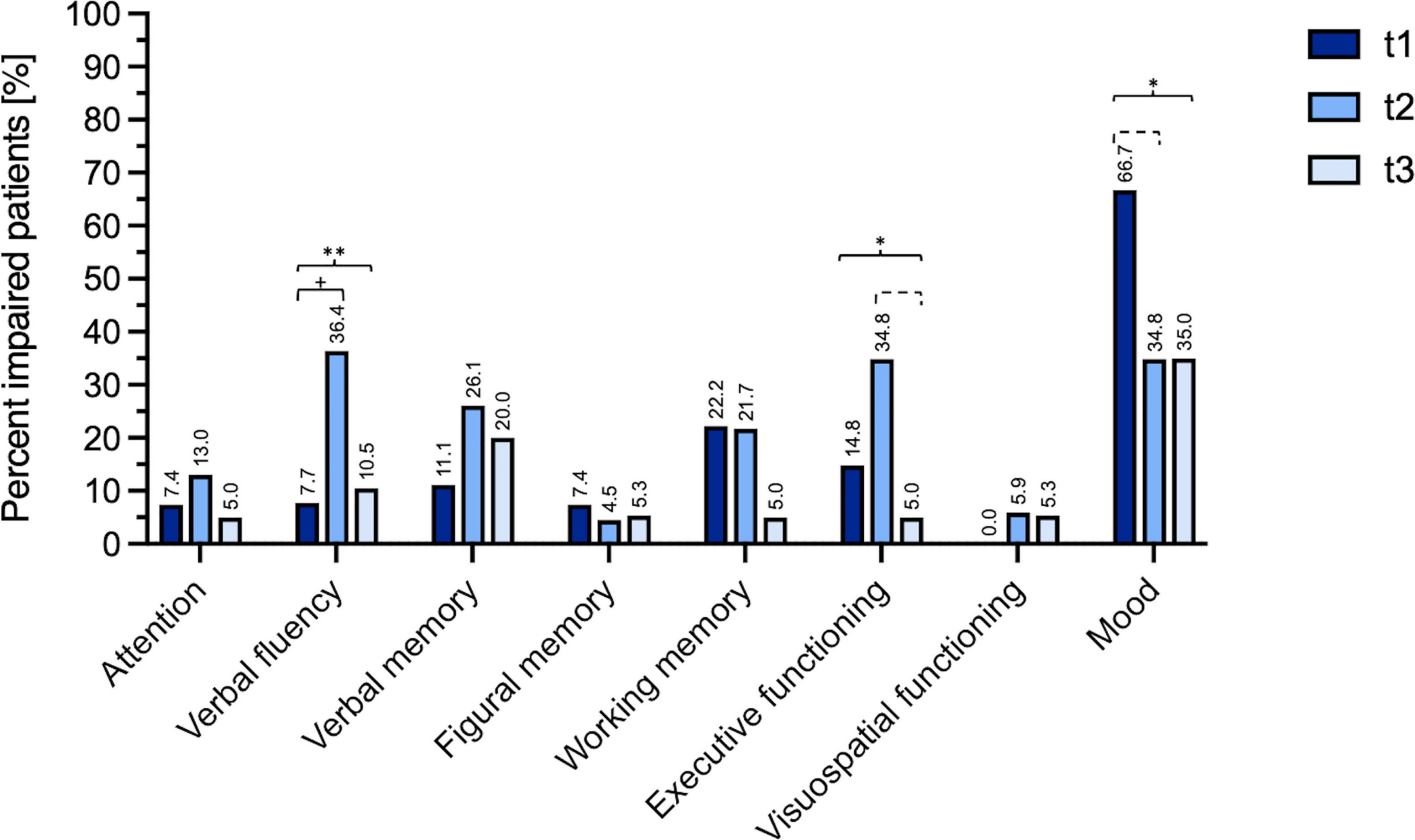
Percent of impaired patients at T1 (preoperatively), T2 (after a median follow-up period of 4.1 months) and T3 (after a median follow-up period of 18.3 months). Long brackets mark results of the Cochran’s analysis (*=p < 0.05, **=p < 0.01), small brackets mark the *post-hoc* tests performed for serial follow up data (+=p < 0.05, dashed bracket p < 0.1). Complete data with Cochran Q’s and *post-hoc* tests for all domains is available as supplementary material (**Supplementary Table 1**).

For each neurocognitive domain, the percentage of patients impaired is presented in **Figure 1**. Moreover, a detailed table with Cochran Q’s and *post-hoc* tests for all domains is available as supplementary material (**Supplementary Table 1**).

### Neurocognitive Performance – Individual Changes

**Figure 2:**
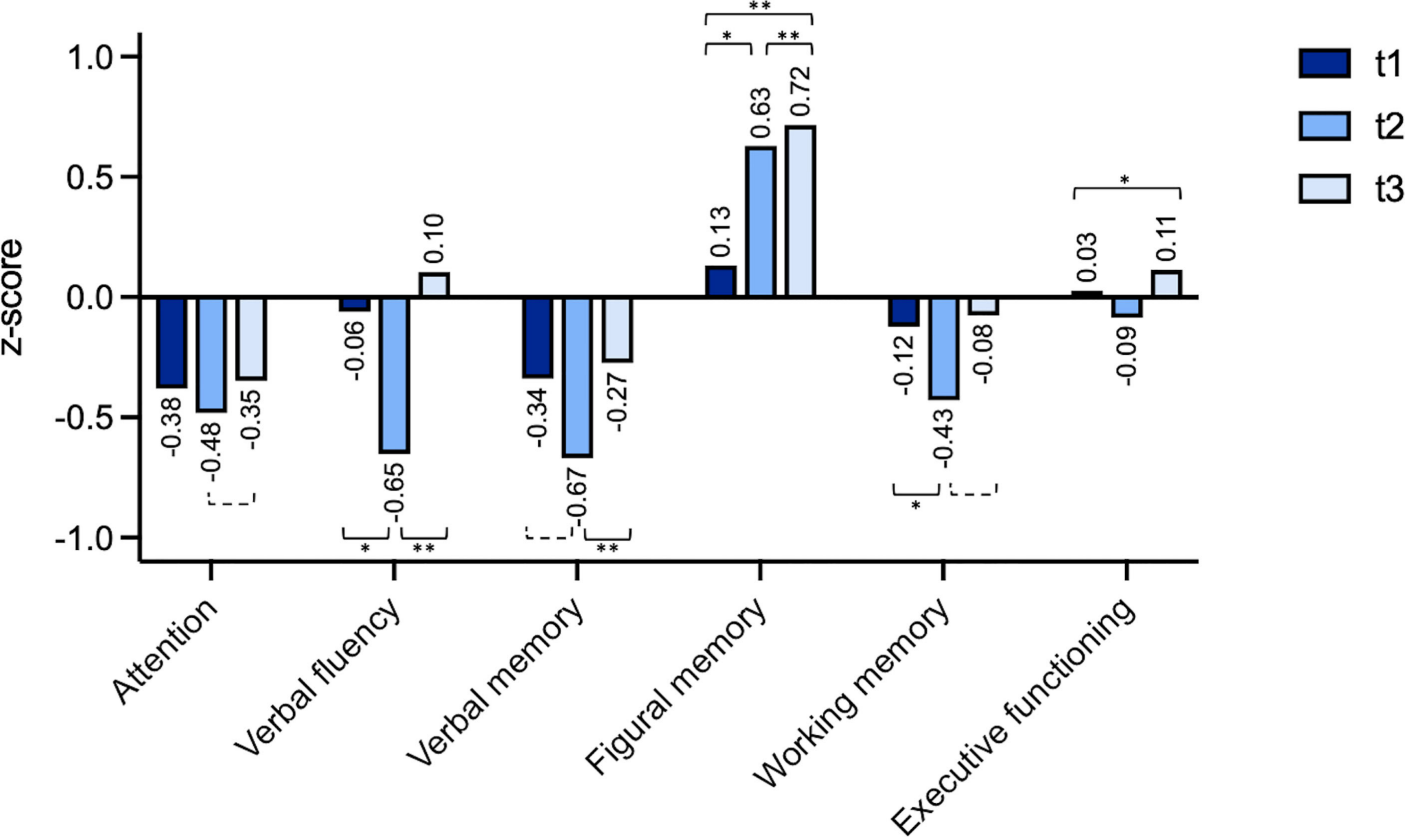
Individual changes (z-scores) t1 (preoperatively), t2 (after a median follow-up period of 4.1 months) and t3 (after a median follow-up period of 18.3 months). The domain “visuospatial functioning” was excluded because the underlying test has no variance of the standardized value in the performance range rated as unimpaired. *=p < 0.05, **=p < 0.01, dashed bracket p < 0.1.

Comparing the mean z-scores, for none of the domains the cut-off of -1.5, defining neurocognitive impairment, was reached (**Figure 2**). Considering observed differences over time and applying a change in z-score > ± 1 as cut-off for a significant change in cognitive performance, a trend of improved neuro-cognitive functioning was found for attention between t3 and t2 (t (14)=2.03, p=.062).

For verbal fluency patients showed a significant worsening for t2 vs. t1 (t (21)=2.82, p=.010 as well as a significant improvement for t3 vs. t2 (t (14)=5.20, p=<.001). For verbal memory patients showed significant improvement for t3 vs. t2 (t (14)=3.41, p=.004). For figural memory patients showed a significant improvement after surgery for t2 vs. t1 (t (21)=2.59, p=.017), t2 vs. t3 (t (12)=3.33, p=.006) and overall comparing t3 vs. t1 (t (18)=3.37, p=.003). For working memory there was a significant worsening for t2 vs. t1 (t (21)=2.67, p=.014) and a trend for improvement comparing t3 vs. t2 (t (14)=2.03, p=0.061).

A detailed table with student t-tests for all domains is available in the supplementary material (**Supplementary Table 2**).

## Discussion

Diagnosis as well as treatment of gliomas represent a significant strain in patients’ lives. The confrontation with a life-threatening disease entails a serious psychological burden for both the patients and their relatives (31). Moreover, tumor- and therapy-related impairments of neurologic and neurocognitive functions often not only decrease patients´ quality of life, but also immediately influence their working ability, subsequently, their financial situation and, most importantly, their social life. Therefore, maintenance or even improvement of patients´ neurologic and neurocognitive functions has to be the utmost aim of glioma patients´ treatment.

We could demonstrate that an individual therapy of glioma patients allows them to return to or even improve their preoperative conditions compared with baseline in the long-term, despite a deterioration of most cognitive functions in the short-term (compare 4). First and foremost, no significant changes were observed in patients´ verbal memory, although proportionally many tumors were located in the temporal lobe (41%), with surgery in the temporal lobe being known to negatively affect verbal memory (32, 33). Nevertheless, to our clinical experience these patients frequently report on short-term memory difficulties. That this experience is not mirrored in the results of the present study might be due to the fact that the respective z-values all ranged between 0 and -0.9, not exceeding the cut off value of -1.5. Thus, at first sight, patients might not have had a relevant deficit in the corresponding domains, neither pre- nor postoperatively. However, despite not crossing the cut off values, z-scores differed significantly over time for the domain verbal fluency, verbal memory, and working memory, demonstrating that patients´ neurocognitive functions change individually in the context of their performance. Therefore, in practice, we recommend both approaches: On the one hand, a development of deficits should be monitored in order to define disturbances relevant to everyday life. On the other hand, patients´ individual changing clinical conditions should be considered in order to recognize changes in their individual framework. Each individual patient´s functions change differently over time, so that a patient-centered, individual assessment and refraining from rigid cut-off values is recommended.

Several measures may have positively influenced the present patients´ neurocognitive outcome and their quality of life, all above the applied neurosurgical technique of awake surgery. A plethora of previous studies has provided evidence that awake surgery is the method of choice for achieving maximal EOR while preserving patients´ neurologic and neurocognitive functions (9–11). Accordingly, GTR and STR could be achieved in 55% and 22% of patients, respectively, with only a small percentage of all patients presenting neurocognitive impairments after a median follow-up period of 18.3 months (t3).

Another prognostic favorable aspect was patients´ excellent preoperative KPS. All patients reported on normal daily activity and were presumably asymptomatic at the moment of presentation in our department and prior to surgery – despite a recent first epileptic seizure having led to tumor diagnosis in 18 (66.7%) patients. The fact that only detailed neurocognitive evaluation revealed cognitive impairments in patients presenting with a KPS of 100% suggests the importance of preoperative assessment, particularly with regard to longitudinal evaluation. Likewise, patients presented a median KPS of 100% also at long-term follow-up, confirming the effectiveness of individual glioma therapy.

Finally, cognitive rehabilitation might have positively influenced patients´ outcome. As demonstrated by a randomized controlled trial, rehabilitation with therapist-guided cognitive training significantly improves patients´ cognitive functions (34). Adequate rehabilitation, especially with regard to speech therapy and cognitive rehabilitation, had been initiated in all our patients immediately after surgery, however, whether and to which extent they pursued rehabilitation therapy in the outpatient setting over time could not be derived from patients´ records. Nevertheless, the premise of cognitive rehabilitation achieving optimal outcomes is meticulous assessment of changes in patients´ neurocognitive performance over time. We therefore strongly recommend glioma patients´ neurocognitive evaluation by a clinical neuropsychologist pre-, peri- and postoperatively. Through appropriate cognitive rehabilitation patients´ return to work, their family life as well as social life will be positively influenced, which in turn will be reflected in higher quality-adjusted life-years and lowered economic burden (35).

The results of the present study are only partially in line with the previously published literature, most obviously due to their heterogeneity of inclusion criteria and the difference of time-intervals between neurocognitive assessments. In the most recent review on supratotal resection of high-grade glioma Tabor et al. reported on a decline in all neurocognitive domains immediately after surgery with return to baseline after a follow-up period of 1 to 4 months, with the exception of memory (36). Another meta-analysis on neuro-cognition after glioma surgery, including both low- and high-grade glioma patients, reported on improved language, attention and memory already in the immediate postoperative period, whereas executive function showed sustained decline also at long-term follow-up 3 to 6 months after surgery (37). A third review by Satoer et al. equally observed a decline in most cognitive domains in the immediate postoperative phase, but found no general significant neurocognitive changes after further 3 to 12 months, with the exception of three reports on improvements in language, memory, attention and/or executive function (38).

However, the present study identified improvements in all neurocognitive domains at long-term follow-up, both compared to preoperative baseline as well as the short-term postoperative phase. Of particular note, we conducted long-term follow-up examinations after a median period of 18.3 months, whereas previous studies evaluating neurocognitive changes over the postoperative period reported on long-term neurocognitive assessments 3 to 6 months after surgery (32, 39–42). By contrast, we defined neurocognitive assessments within the first 9 postoperative months to fall into short-term evaluations (with the median coming up to 4.1 months), since we observed a significant change in patients´ neurocognitive performances at this time point during their course of disease. Considering this relatively “late” first postoperative follow-up evaluation the percentage of cognitively impaired patients was quite high at this time point, however, the respective z-scores all ranged far above -1.5, confirming the mild character of patients´ neurocognitive deterioration.

The results in the present study might, however, have been confounded by the presence of epileptic seizures preoperatively, by ongoing anti-epileptic therapy thereafter and adjuvant oncologic treatment. While all patients were seizure-free after tumor surgery, 22 (81%) patients continued using anti-epileptic drugs, most probably to maintain or return to an independent and fulfilled social and working life (43). However, in contrast to previous studies (44) anti-epileptic therapy did not result in a relevant deterioration of cognition in our patients. We therefore advocate to achieve seizure freedom by the use of “newer” anti-epileptic drugs such as Lacosamide or Levetiracetam, which were applied to our patients, and which have been shown to improve neurocognition and behavior through its effect on seizure control (45, 46).

Regarding adjuvant oncologic treatment, 21 (77.8%) patients of the present cohort underwent combined chemo-radiotherapy. Although several studies have reported on cognitive decline affecting all domains already 6 months following adjuvant treatment (47, 48), especially after radiotherapy (49), we did not observe a correlation of the present patients´ neurocognitive performance and adjuvant treatment.

## Limitations

This study had some limitations. First, follow-up neurocognitive data sets were not available for all patients. Due to patients’ refusal to undergo further postoperative cognitive evaluation, longitudinal follow-up evaluations were only possible in 16 patients. Unfortunately, the number of patients did not allow for a multivariant analysis which would have been necessary to confirm that neurocognitive outcome is influenced by tumor characteristics, surgical and seizure outcome as well as adjuvant treatment.

Thus, the heterogeneity of patient cohorts may have biased presented results. A valid objection is, that low grade and high grade gliomas were mixed. Since the present study aimed at analyzing the longitudinal neurocognitive outcome after awake surgery depending on glioma localizations (IFG, ATL, dMTG/STG) the analysis of both, low and high grade glioma patients was accepted. Moreover, it is likely that only patients with relatively good clinical performance biased our results. On the one hand, only patients with tumors being amenable to a great extent of tumor resection were included into our study; on the other hand, only patients who were willing and able to undergo long-term follow-up cognitive evaluations were included and might have possibly caused an overestimation of clinical and neurocognitive results.

Moreover, data on patients´ individual physical, neurocognitive, linguistic and occupational therapies were not evaluated in detail, since detailed records on these therapies were incomplete. Nevertheless, since 17 of 27 patients underwent postoperative rehabilitation therapy in highly-specialized neurological rehabilitation hospitals, and since those other patients who displayed minor deficits either regarding neurocognition and/or language function and/or fine motor function deficits were recommended to undergo neurocognitive, linguistic and occupational therapies in the outpatient setting, we presume that nearly all patients received one or more types of postoperative therapies.

For the analysis of the neurocognitive data the calculation of reliable change indices (RCIs) is also possible. The rationale for not using this method came from the fact that they are rarely calculated in clinical practice and data would have not been comparable. Another lack in neurocognitive testing is not using basal language tests [e.g. Aachen Aphasia Test (50) or Boston Naming Test (51)] which should be considered for future studies. Regarding patients´ consistently good KPS, it should be discussed that neurocognitive deficits, which were assessed with detailed psychometric tests, did not seem to be functionally relevant in simple everyday situations (such as clinical rounds). Further evaluation in a bigger cohort of patients is therefore mandatory, in order to allow for further meaningful correlations of clinical, surgical, and cognitive data.

## Conclusion

Awake surgery in patients with eloquently located gliomas allows for an excellent functional outcome and seizure-freedom in the long-term course. With regard to neurocognitive assessment, individual patients´ functional courses of disease need to be considered in addition to cut-offs values. In light of these favorable outcomes the results of the present study may help neurosurgeons and neuro-oncologists in deciding on personalized therapeutic strategies and in counselling of glioma patients.

## Data Availability Statement

The raw data supporting the conclusions of this article will be made available by the authors, without undue reservation.

## Ethics Statement

The studies involving human participants were reviewed and approved by Ethics committee University Hospital Frankfurt. The patients/participants provided their written informed consent to participate in this study. Written informed consent was obtained from the individual(s) for the publication of any potentially identifiable images or data included in this article.

## Author Contributions

M-TF, MB, and SR conceived the presented idea and developed the experimental design. Neurocognitive assessments were conducted by IL, MB, and NC. KF was involved in the neuropathological workup. MV participated regarding neurooncological issues. Intra-operative testing during awake surgery was performed by IL and CK. MR provided advice on neuroradiological issues. M-TF, MB, and SR prepared the submitted manuscript. All authors contributed to the article and approved the submitted version.

## Conflict of Interest

The authors declare that the research was conducted in the absence of any commercial or financial relationships that could be construed as a potential conflict of interest.

## Publisher’s Note

All claims expressed in this article are solely those of the authors and do not necessarily represent those of their affiliated organizations, or those of the publisher, the editors and the reviewers. Any product that may be evaluated in this article, or claim that may be made by its manufacturer, is not guaranteed or endorsed by the publisher.

## Abbreviations

AED, antiepileptic drugs; EOR, extent of resection; CI, confidence interval; GBM, Glioblastoma; GTR, gross total resection; HADS, Hospital Anxiety and Depression Scale; HGG, High grade glioma; IDH-1, isocitrate dehydrogenase 1; IQR, interquartile ranges; KPS, Karnofsky performance score; LGG, low grade glioma; MMSE, mini-mental state examination; PD, progressive disease; PR, partial resection; SD, stable disease; STR, subtotal resection.
