# Supplementary material for: Neurocognitive Outcome and Seizure Freedom After Awake Surgery of Gliomas
Source: Front Oncol. 2022 Apr 7;12:815733. doi: 10.3389/fonc.2022.815733 (PMC9023117; doi:10.3389/fonc.2022.815733)
Supplement: Supplementary file 1 [file Table_1.docx]

|  | n | Cochran's Q | p-value | n Impaired | | % Impaired | |  | n | McNemar |
| --- | --- | --- | --- | --- | --- | --- | --- | --- | --- | --- |
|  |  |  |  | no | yes | no | yes |  |  | p-value |
| Attention |  |  |  | 15 | 1 | 93.8 | 6.3 | t1 vs. t2 | 16 | 0.500 |
|  | 16 | 4.000 | 0.135 | 13 | 3 | 81.3 | 18.8 | t2 vs. t3 | 16 | 0.500 |
|  |  |  |  | 15 | 1 | 93.8 | 6.3 | t1 vs. t3 | 16 | 1.000 |
| Verbal fluency |  |  |  | 15 | 0 | 100 | 0 | t1 vs. t2 | 15 | 0.031* |
|  | 15 | 9.333 | 0.009** | 9 | 6 | 60 | 40 | t2 vs. t3 | 15 | 0.125 |
|  |  |  |  | 13 | 2 | 86.7 | 13.3 | t1 vs. t3 | 15 | 0.500 |
| Verbal memory |  |  |  | 15 | 1 | 93.8 | 6.3 | t1 vs. t2 | 16 | 0.375 |
|  | 16 | 2.571 | 0.276 | 12 | 4 | 75 | 25 | t2 vs. t3 | 16 | 1.000 |
|  |  |  |  | 12 | 4 | 75 | 25 | t1 vs. t3 | 16 | 0.375 |
| Figural memory |  |  |  | 16 | 0 | 100 | 0 | t1 vs. t2 | 14 | 1.000 |
|  | 14 | 1.000 | 0.607 | 14 | 1 | 93.3 | 6.7 | t2 vs. t3 | 14 | 1.000 |
|  |  |  |  | 14 | 1 | 93,3 | 6.7 | t1 vs. t3 | 14 | 1.000 |
| Working memory |  |  |  | 12 | 4 | 75 | 25 | t1 vs. t2 | 16 | 1.000 |
|  | 16 | 4.667 | 0.097 | 13 | 3 | 81.3 | 18.8 | t2 vs. t3 | 16 | 0.500 |
|  |  |  |  | 15 | 1 | 93.8 | 6.3 | t1 vs. t3 | 16 | 0.250 |
| Executive functioning |  |  |  | 14 | 2 | 87.5 | 12.5 | t1 vs. t2 | 16 | 0.219 |
|  | 16 | 6.000 | 0.049* | 10 | 6 | 62.5 | 37.5 | t2 vs. t3 | 16 | 0.063^+^ |
|  |  |  |  | 15 | 1 | 93.8 | 6.3 | t1 vs. t3 | 16 | 1.000 |
| Visuospatial functioning |  |  |  | 16 | 0 | 100 | 0 | t1 vs. t2 | 10 | n.c. |
|  | 10 | 2.000 | 0.368 | 10 | 0 | 100 | 0 | t2 vs. t3 | 10 | 1.000 |
|  |  |  |  | 14 | 1 | 93.3 | 6.7 | t1 vs. t3 | 10 | 1.000 |
| Mood |  |  |  | 5 | 11 | 31.3 | 68,.8 | t1 vs. t2 | 16 | 0.070 |
|  | 16 | 7.000 | 0.030* | 11 | 5 | 68.8 | 31.3 | t2 vs. t3 | 16 | 0.500 |
|  |  |  |  | 9 | 7 | 56.3 | 43.8 | t1 vs. t3 | 16 | 0.219 |

**Supplement Table 1:** Cochran’s Q Tests, post-hoc McNemar tests. Only patients with neurocognitive assessment at all time points were included in the analysis. Abbreviation n.c. = not comparable.

^+^=p<0.1, *=p<0.05, **=p<0.01.
