# Supplementary material for: Neurocognitive Outcome and Seizure Freedom After Awake Surgery of Gliomas
Source: Front Oncol. 2022 Apr 7;12:815733. doi: 10.3389/fonc.2022.815733 (PMC9023117; doi:10.3389/fonc.2022.815733)
Supplement: Supplementary file 2 [file Table_2.docx]

|  |  | n | z score | |  | n | t-test | |
| --- | --- | --- | --- | --- | --- | --- | --- | --- |
|  |  |  | mean | sd |  |  | t-value | p-value |
| Attention | t1 | 27 | -0.380 | 0.492 | t1 vs. t2 | 22 | 0.997 | 0.330 |
|  | t2 | 22 | -0.481 | 0.452 | t2 vs. t3 | 15 | -2.027 | 0.062^+^ |
|  | t3 | 20 | -0.348 | 0.324 | t1 vs. t3 | 20 | -0.375 | 0.712 |
| Verbal fluency | t1 | 26 | -0.060 | 0.732 | t1 vs. t2 | 22 | 2.824 | 0.010* |
|  | t2 | 22 | -0.652 | 1.083 | t2 vs. t3 | 15 | -5.200 | 0.000** |
|  | t3 | 19 | 0.104 | 0.852 | t1 vs. t3 | 19 | -1.197 | 0.247 |
| Verbal memory | t1 | 27 | -0.338 | 0.736 | t1 vs. t2 | 22 | 2.062 | 0.052 |
|  | t2 | 22 | -0.669 | 0.742 | t2 vs. t3 | 15 | -3.407 | 0.004** |
|  | t3 | 20 | -0.274 | 0.770 | t1 vs. t3 | 20 | -0.466 | 0.646 |
| Figural memory | t1 | 27 | 0.132 | 0.725 | t1 vs. t2 | 21 | -2.592 | 0.017** |
|  | t2 | 21 | 0.629 | 0.642 | t2 vs. t3 | 13 | -3.330 | 0.006** |
|  | t3 | 19 | 0.716 | 0.750 | t1 vs. t3 | 19 | -3.365 | 0.003** |
| Working memory | t1 | 27 | 0.122 | 0.866 | t1 vs. t2 | 22 | 2.668 | 0.014** |
|  | t2 | 22 | 0.429 | 0.983 | t2 vs. t3 | 15 | -2.033 | 0.061^+^ |
|  | t3 | 20 | 0.076 | 0.787 | t1 vs. t3 | 20 | -0.091 | 0.928 |
| Executive functioning | t1 | 27 | 0.026 | 0.549 | t1 vs. t2 | 20 | 0.473 | 0.641 |
|  | t2 | 20 | 0.086 | 0.893 | t2 vs. t3 | 13 | -0.897 | 0.387 |
|  | t3 | 20 | 0.113 | 0.505 | t1 vs. t3 | 20 | -0.629 | 0.537 |
| Visuospatial functioning | t1 | 27 | 0.994 | 0.000 | t1 vs. t2 | 17 | -0.157 | 0.877 |
|  | t2 | 14 | 0.994 | 0.000 | t2 vs. t3 | 10 | 1.000 | 0.343 |
|  | t3 | 19 | 1.065 | 0.306 | t1 vs. t3 | 19 | -0.485 | 0.633 |

**Supplement Table 2:** Comparison of z-score means, post-hoc t-tests.

^+^=p<0.1, *=p<0.05, **=p<0.01
